# Contextual Modulation of Vocal Behavior in Mouse: Newly Identified 12 kHz “Mid-Frequency” Vocalization Emitted during Restraint

**DOI:** 10.3389/fnbeh.2016.00038

**Published:** 2016-03-09

**Authors:** Jasmine M. S. Grimsley, Saloni Sheth, Neil Vallabh, Calum A. Grimsley, Jyoti Bhattal, Maeson Latsko, Aaron Jasnow, Jeffrey J. Wenstrup

**Affiliations:** ^1^Department of Anatomy and Neurobiology, Northeast Ohio Medical UniversityRootstown, OH, USA; ^2^Department of Psychological Sciences, Kent State UniversityKent, OH, USA

**Keywords:** vocalization, stress, restraint, isolation, mouse, context

## Abstract

While several studies have investigated mouse ultrasonic vocalizations (USVs) emitted by isolated pups or by males in mating contexts, studies of behavioral contexts other than mating and vocalization categories other than USVs have been limited. By improving our understanding of the vocalizations emitted by mice across behavioral contexts, we will better understand the natural vocal behavior of mice and better interpret vocalizations from mouse models of disease. Hypothesizing that mouse vocal behavior would differ depending on behavioral context, we recorded vocalizations from male CBA/CaJ mice across three behavioral contexts including mating, isolation, and restraint. We found that brief restraint elevated blood corticosterone levels of mice, indicating increased stress relative to isolation. Further, after 3 days of brief restraint, mice displayed behavioral changes indicative of stress. These persisted for at least 2 days after restraint. Contextual differences in mouse vocal behavior were striking and robust across animals. Thus, while USVs were the most common vocalization type across contexts, the spectrotemporal features of USVs were context-dependent. Compared to the mating context, vocalizations during isolation and restraint displayed a broader frequency range, with a greater emphasis on frequencies below 50 kHz. These contexts also included more non-USV vocal categories and different vocal patterns. We identified a new Mid-Frequency Vocalization, a tonal vocalization with fundamental frequencies below 18 kHz, which was almost exclusively emitted by mice undergoing restraint stress. These differences combine to form vocal behavior that is grossly different among behavioral contexts and may reflect the level of anxiety in these contexts.

## Introduction

In acoustic communication, vocalizations carry information about the state of a caller and influence the state of a listener. A full understanding of acoustic communication systems requires an in-depth assessment relating vocalizations to caller and listener states. This study relates vocalizations to the state of calling mice. Mice have become an important model of vocal behavior and social communication, with research focusing both on the communication system within the brain and on the use of vocalizations as biomarkers of animal state in health and disease (Panksepp et al., 2007; Bishop and Lahvis, 2011; Lahvis et al., 2011; Wöhr et al., 2011). However, it has been unclear what functions are served by acoustic communication in mice. Even the most common mouse vocal category, the ultrasonic vocalization (USV), may have multiple functions since it is emitted in a wide range of positive and negative contexts (see Arriaga, 2014). Further, using vocalizations to investigate diseases requires an understanding of how these vocalizations vary depending on the internal state of healthy animals.

The vast majority of studies of mouse vocal behavior have focused on USVs (>20 kHz), emitted by male mice during courtship and mating (Whitney and Nyby, 1979; Maggio et al., 1983), but also by both male and female adults during other social interactions (Maggio and Whitney, 1985; Grimsley et al., 2011). USVs are also the most commonly emitted vocalization by mouse pups (Liu et al., 2003; Scattoni et al., 2009; Grimsley et al., 2011). There is clear evidence that these may be modulated as a function of development (Liu et al., 2003; Grimsley et al., 2011) and courtship and mating (Chabout et al., 2015). This study adds to the understanding of mouse USVs by assessing differences that result from isolation and the stress of restraint. We hypothesize that the modulation of mouse USV structure has the potential to convey information about the state of a calling animal.

Mice emit at least two other categories of vocalizations with very different spectral characteristics. We have referred to these as low frequency harmonic (LFH) vocalizations and “noisy” vocalizations (Grimsley et al., 2011). They are typically excluded from most studies of mouse vocal behavior, but may provide different or additional information about a calling animal's internal state. The LFH vocalization is a harmonic complex with power well within the audible range; this is the “squeak” commonly associated with mice. Power in the LFH vocalization is broadband, with harmonics starting near 5 kHz and extending upwards of 100 kHz. Like USVs, the LFH vocalization is emitted in a variety of negative behavioral contexts, including pain (Whitney and Nyby, 1983), agitation (Grimsley et al., 2013), fighting (Houseknecht, 1968; Gourbal et al., 2004), and by females during courtship (Sales, 1972b; Grimsley et al., 2013). There is no difference in the spectrotemporal features of LFH vocalizations emitted by females in these contexts, however the meaning of these vocalizations to males appears to depend on cues from other sensory modalities (Grimsley et al., 2013). In sexually naïve mice, the LFH vocalization is so strongly aversive that it can be used as an unconditioned stimulus within classical conditioning studies (Chen et al., 2009). The significance of the noisy vocalizations is not understood. For each of these vocalizations, we hypothesized that stressful situations may alter their emission by mice.

Our principal goal in this study was to assess if and how mice modify their vocalizations as a function of behavioral context and associated internal state. We used restraint to create a stressful context, as restraint reliably induces stress in mice (Pare and Glavin, 1986). We hypothesized that vocalizations produced by male mice that are stressed, isolated, or mating would have different spectrotemporal characteristics and emission patterns. The results show that mice robustly emit different vocal categories depending on the behavioral context, and we identify a new vocalization commonly emitted by animals under restraint that is within the human hearing range.

## Methods

This study compared physiological, behavioral, and vocal differences resulting from restraint and social isolation. All procedures were approved by the Institutional Animal Care and Use Committee at the Northeast Ohio Medical University. A total of 77 adult CBA/CaJ male mice between 100 and 190 days old were used in this study. CBA/CaJ mice are a standard control strain in auditory research due to their sensitive hearing thresholds that are maintained up to at least 39 weeks (Zheng et al., 1999). To avoid issues arising from comparisons across litters (Zorrilla, 1997), we used litter-matched pairs. Mice were housed on a reversed, 12-h light/dark cycle. All experiments were performed during the dark portion of the cycle, starting 2–3 h into the dark period.

### Experimental groups

Mouse vocalizations were recorded within a single-walled acoustic chamber (Industrial Acoustics, New York, NY) lined with anechoic foam on a table covered in white laboratory paper. Acoustic signals were recorded by an ultrasonic condenser microphone (Avisoft Bioacoustics). The recording system was flat (±3 dB) between 20 and 140 kHz, with a low-frequency roll-off of 12 dB per octave. The microphone signal was digitized at 500 kHz and 16-bit depth. Vocalizations were obtained under several conditions described below and outlined in Table 1.

**Table 1:** **The number of animals included in each experimental group**.

**Context**						
**Experiment**	**Isolation**	**Jacket restrained**	**Tube restrained**	**Headpost restrained**	**Headpost isolated**	**Mating**	**Total**
Corticosterone	10	6	4				20
Behavior	8	8					16
Vocal recording	14	14	5	4	4	^*^41	41
Total	32	28	9	4	4	^*^41	77

* *The vocal behavior of all males was first recorded during a mating interaction. These animals' vocalizations were then recorded in one other context*.

#### Male-female interaction

For all animals in the vocal recording experiment (*n* = 41), vocalizations were initially recorded during the first hour of a mating interaction that occurred on 3 consecutive days (see Table 1). The estrus cycle of the females in these interactions was not controlled, potentially increasing variability in the calling rate of males. Mice were placed in a clean, standard mouse home cage (length = 28 cm, width = 20 cm, height = 15 cm). The microphone was situated centrally 15 cm above the cage floor. USVs emitted in male-female interactions are thought to be emitted almost exclusively by the male mouse (Whitney and Nyby, 1979; Maggio et al., 1983). Conversely, LFH vocalizations are typically emitted by female mice when males attempt to mount them (Sales, 1972a; Grimsley et al., 2013). In this study only 353 overlapping vocalizations were recorded from a total of 80,579 vocalizations; all instances included a combination of a LFH and USV. Because it is not possible to determine which vocalization was emitted by the male or the female, both vocalizations were omitted from further analysis.

#### Isolation in a spherical arena

In a subset of animals (*n* = 14), from which vocalizations had previously been recorded during mating, vocalizations were recorded when they were isolated for 2-h in a novel spherical arena (volume = 7.5 liter, diameter = 24 cm) and placed on a raised, round platform (diameter = 14 cm, height = 2 cm) covered with a layer of absorbent material. Animals underwent this isolation on 3 consecutive days. The recording microphone was positioned 8 cm above the animal, pointing downward. These mice were litter-matched to the *jacket restraint* group.

#### Jacket restraint

In a subset of animals (*n* = 14), from which vocalizations had previously been recorded during mating, vocalizations were obtained while under restraint. These mice were litter-matched to the *isolation in a spherical arena* group. These animals were placed in jackets that were suspended from the top of the same spherical arena used in the isolation condition. The feet of these animals did not touch the floor. The recording microphone was positioned 8 cm above the animal pointing downward.

Although the restraining jacket did not fit tightly over the chest of the mice, and was unlikely to mechanically affect vocalizations, we used two additional forms of restraint to ensure that vocalization changes were not due to chest constriction.

#### Tube restraint

In a subset of animals (*n* = 5), from which vocalizations had previously been recorded during mating, vocalizations were obtained while the mouse was placed in a narrow tube (diameter = 3 cm, length = 11 cm). Although the tube was narrow, some animals were able to turn around within it. Wire mesh was placed over the tube ends and recordings were made simultaneously from both ends of the tube. Here, sounds were analyzed from the channel that recorded the vocalization at the greatest intensity. Animals were restrained for 2-h.

#### Headpost restraint

In a subset of animals (*n* = 4), from which vocalizations had previously been recorded during mating, vocalizations were obtained while the mouse was restrained by a headpost mounted to the skull, a method typically used for restraint during electrophysiology studies (described by Muniak et al., 2012). Briefly, animals were anesthetized to effect with isoflurane (2–4%, Abbott Laboratories, Abbott Park, IL). Fur on the skin overlying the skull was removed using depilatory lotion. A midline incision was made to bilaterally expose the frontal and parietal plates. A small metal post (height = 1.6 cm, width = 0.2 cm) was secured to the skull over bregma using dental cement. Subsequently, topical local anesthetic (Lidocaine, Johnson amd Johnson, Skillman, NJ) and antibiotic cream (Neosporin, Ferndale laboratories, Ferndale, MI) were applied to the surgical area and animals were placed in a clean, heated cage to recover. Animals were left to recover for 3 days prior to testing. Animals were restrained in a custom stereotaxic device for 2-h. During sound recording, a microphone was positioned 8 cm in front of the animal's mouth. These mice were litter-matched to the *headpost with isolation in spherical arena* group.

#### Headpost with isolation in spherical arena

In a subset of animals (*n* = 4) from which vocalizations had previously been recorded during mating, vocalizations were obtained while isolated in the spherical arena. These mice were litter-matched to the *headpost restraint* group. Mice in this group underwent the same surgery to implant a headpost as described above, but were not restrained during recording. These animals were isolated for 2-h within the same arena as was used for the isolation contexts.

### Hormonal assessment of stress

Corticosterone levels were used to compare the levels of stress (Gong et al., 2015) in litter matched pairs of animals undergoing isolation or restraint (*n* = 10 pairs, 4 pairs with headposts). Blood was collected between 3 h and 5 h into the dark part of their cycle. Within 1 min of completion of exposure, animals were rapidly anesthetized with Isoflurane (4%, Abbott Laboratories, Abbott Park, IL) and immediately injected with a lethal dose of barbiturate (Fatal Plus). Once animals were deeply anesthetized, the chest cavity was opened and blood sample was removed from the heart with a syringe and hypodermic needle. Blood was clotted for 1 h, after which the clot was removed and discarded. The remaining liquid was centrifuged at 4°C for 1 h at 350 rpm, after which serum was removed and stored at −80°C until it was processed. Serum from all animals was processed simultaneously. Serum corticosterone was measured using Enzo Life Sciences corticosterone enzyme-linked immunosorbant assay (EIA) kit (Farmingdale, NY; catalog number ADI-900-097), according to the manufacturer's instructions. Each sample was measured in duplicate. The cross reactivity for the corticosterone assay was 28.6% for deoxycorticosterone, 1.7% for progesterone, and <0.3% for all other hormones. Inter-assay variability was <10%.

### Behavioral assessments of anxiety

Behavioral assessments of anxiety (light-dark box, marble burying test) were obtained for eight litter-matched pairs (eight mice with jacket restraint, eight mice unrestrained in isolation chamber). Behavioral tests occurred within a single-walled acoustic chamber (Industrial Acoustics, New York, NY) lined with anechoic foam.

The light-dark box was used to measure baseline anxiety levels prior to the first period of jacket restraint or isolation. Stressed/anxious animals spend a greater proportion of the time in the dark (Crawley and Goodwin, 1980). The box, made in-house, (length = 37 cm, width = 30 cm, height = 25 cm) had a darkened section comprising one-third of the arena. Animals could cross between the light and dark regions via a small open doorway (height = 7 cm, width = 4 cm). Initially, each animal was placed in the center of the light portion, then the time spent in each zone was measured for 10 min using DataWave Videobench (DataWave Sciworks, Loveland, CO).

To test for persistent changes in mouse behavior that may be induced by jacket restraint or isolation, animals were re-tested in the light-dark box on the day after the final session of restraint or isolation. Behavior was further assessed the following day using a marble burying test in which 14 marbles were placed on soft bedding on the floor of the test chamber (length = 28 cm, width = 20 cm, height = 15 cm). The position of marbles was photographed before and after a 15-min test session, and the number of marbles at least 75% buried was recorded. Anxious animals typically bury more marbles (Angoa-Perez et al., 2013).

### Analysis of vocal data

Automated thresholds for detection and identification of vocalizations could not be used because animal movement and other background noise was too high. However, manual analysis of all vocalizations (>3000 calls per session) would be very time consuming. We therefore investigated whether the first 100 vocalizations emitted on a single day were representative of the vocalizations emitted within an entire test session. In seven litter-matched pairs, we found no difference in the vocal repertoire for the first 100 vocalizations or those recorded during the entire session. These results are summarized in Appendices A and B. As a result of this analysis, vocalization data presented in the main body of the Results are based on the first 100 vocalizations from each session. In additional analyses, we examined whether the vocal repertoire changes across subsequent test sessions (Appendix A).

**Figure 1:**
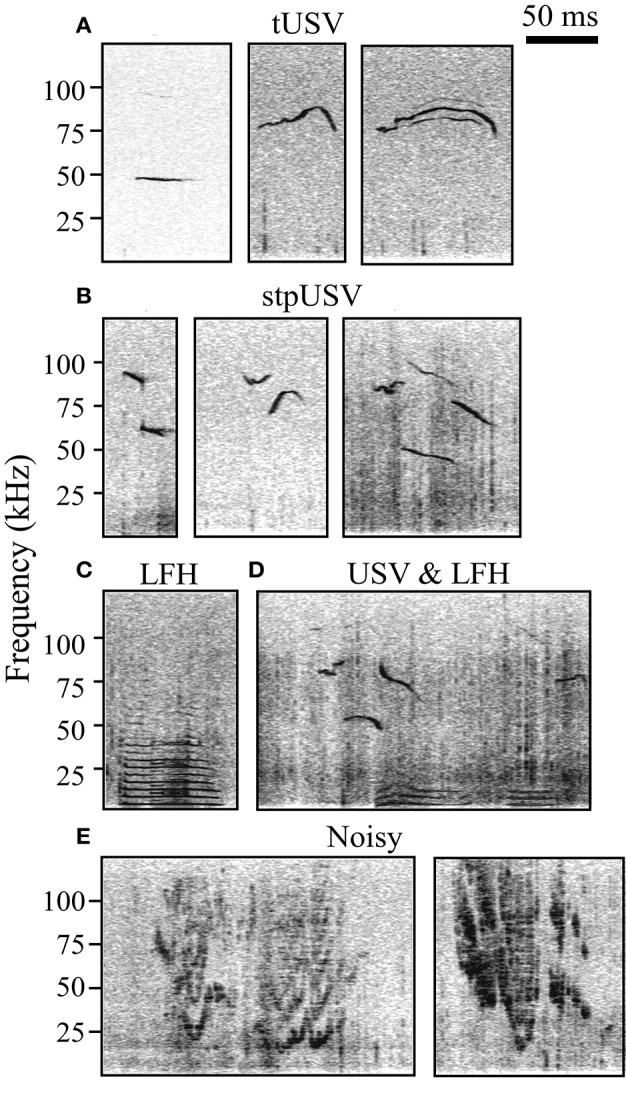
**Spectrograms of established categories of mouse vocalizations. (A)** Tonal ultrasonic vocalizations (tUSV). **(B)** Stepped ultrasonic vocalizations (stpUSV). **(C)** Low frequency harmonic vocalizations (LFH). **(D)** USVs and LFHs emitted during mating/courtship. USVs are likely emitted by the male, while LFHs are likely emitted by the female. **(E)** Noisy vocalizations.

Sound files were analyzed using Avisoft Bioacoustics SAS lab software. Four trained investigators manually added timestamps to spectrograms (Hamming window, FFT length = 1024, frame size = 100%, overlap = 98.43%) indicating the start and end of each vocalization and placed vocalizations into three categories: LFH, Noisy, or USV (Figure 1). LFH vocalizations are characterized by a fundamental below 8 kHz and the presence of multiple harmonics, typically extending into the ultrasonic range (Grimsley et al., 2011). Noisy vocalizations are warbled and broadband with a noisy-harmonic chaotic structure (Grimsley et al., 2011). USVs, emitted in bouts (Maggio and Whitney, 1985; Grimsley et al., 2011), were separated into two subtypes based on the presence or absence of an abrupt frequency step. Tonal USVs (tUSV) do not have abrupt frequency steps, while stepped USVs (stpUSV) have one or more abrupt steps of ≥10 kHz (see Grimsley et al., 2011). We used a nested ANOVA to test for effects of experimenter on the proportion of vocal categories tagged within each test context. There was no main effect of experimenter (*p* = 0.3).

Spectral measurements were computed for each tagged vocalization from spectrograms (Hamming window, FFT length = 1024, frame size = 100%, overlap = 98.43%), with a frequency range restricted to 1–130 kHz. The frequency contour of each vocalization was extracted using Automated Parameter Measurements, a feature of SASLab. The dominant frequency was automatically computed at 8 evenly spaced time points within each vocalization. The mean dominant frequency of each vocalization was computed by averaging the 8 measurements of dominant frequency.

Data were analyzed using the SPSS 17 statistics package. The multiple factors affecting the probabilities of emitting each vocal category by each animal within each context were compared using multi-factor ANOVAs. Multi-factorial ANOVAs were also used to investigate whether restraint had effects on the spectrotemporal features of vocalizations. All *post-hoc* tests used Bonferroni corrections to control for multiple tests. All error bars in figures display 95% confidence intervals.

## Results

We first show that a 2-h period of restraint elevated blood corticosterone levels in mice. We then describe persistent behavioral changes in mice that resulted from repeated bouts of restraint. Finally, we present detailed analyses of vocalizations that revealed gross changes as a result of behavioral context. Additional data that support the experimental approach or provide more in-depth analyses useful to some investigators are presented in Appendices A and B.

### Physiological and behavioral assessments of anxiety

#### Hormonal changes

**Figure 2:**
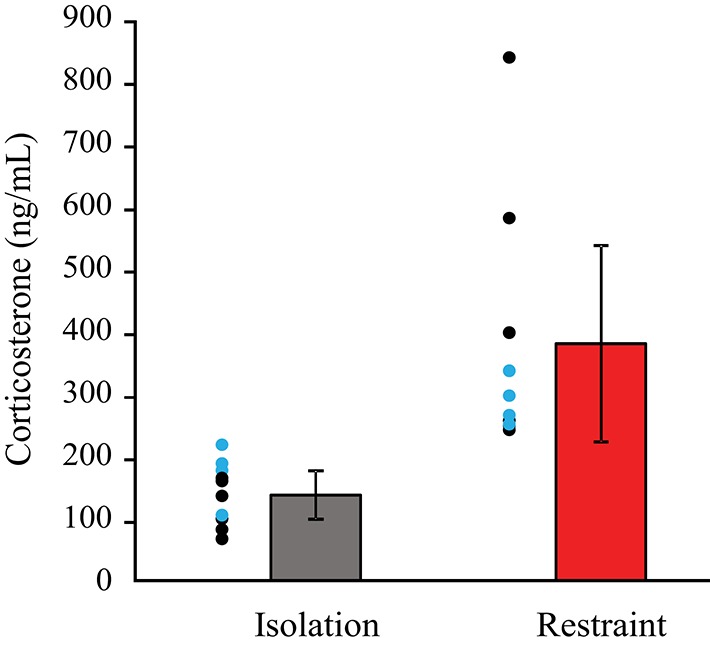
**Blood levels of corticosterone are higher in restrained animals**. Individual circles show data for individual animals: black circles indicate pairs in the jacket/isolation paradigm, blue circles indicate pairs in the headpost/isolation paradigm. Red bars show pooled data across restraint types. Pairwise comparisons revealed that corticosterone levels were significantly higher in restraint (*p* = 0.006), but did not differ between restraint types (*p* = 0.607).

Animals undergoing restraint had significantly higher levels of corticosterone compared to siblings that underwent isolation (Figure 2). This indicates that a brief, 2-h period of restraint results in acute stress in mice. A multivariate ANOVA revealed that there was a significant main effect of context, isolation vs. restraint (*F* = 9.823 *p* = 0.006). Comparison of the partial Eta^2^ showed that a large amount of the variance in corticosterone levels is associated with context (Eta^2^ = 38%), whereas restraint type accounts for only 1.7%. There was no significant main effect of restraint type (*p* = 0.607) and no interaction between the restraint type (headpost vs. jacket) and context (isolation vs. restraint) on the corticosterone levels. *Post-hoc*, pairwise comparisons revealed that corticosterone levels were higher (*p* = 0.006) in animals that underwent restraint. These levels did not differ between restraint types (*p* = 0.607).

#### Behavioral changes

**Figure 3:**
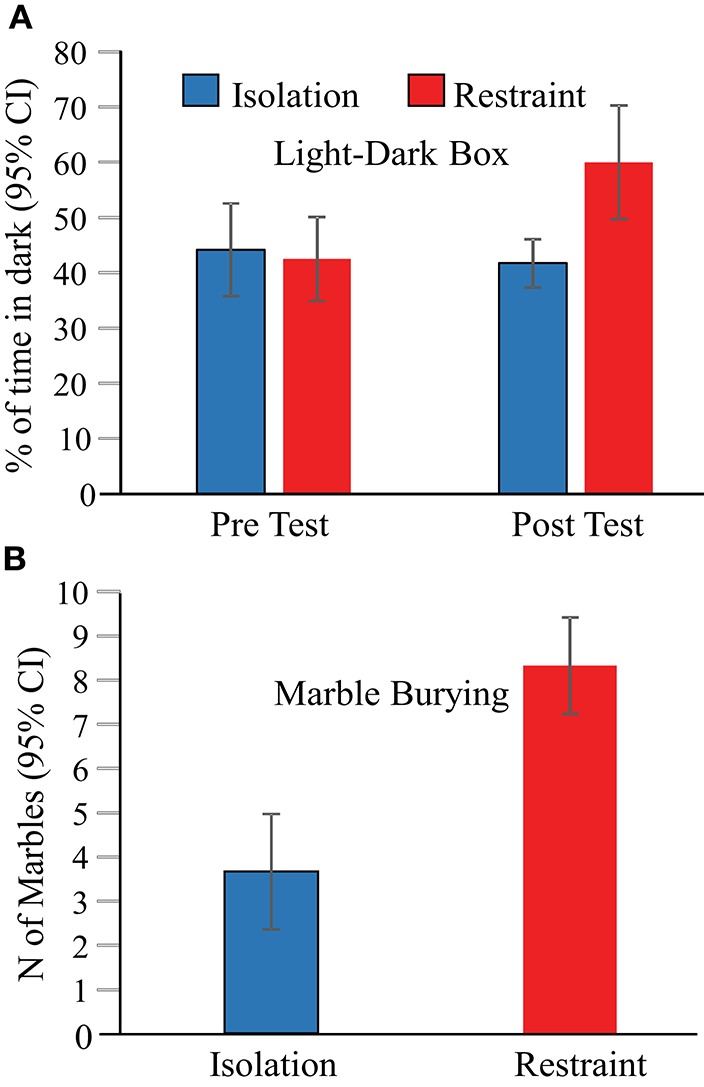
**Behavioral assessments indicate increased anxiety in restrained mice. (A)** Animals undergoing jacket restraint spent more time in the dark after restraint. There was no difference in the time spent in the dark for animals undergoing isolation (blue bars) or between the two groups before restraint (“Pre Test”). **(B)** Animals undergoing restraint buried more marbles than those that underwent isolation.

Behavioral assessments of anxiety were performed at three time points; (1) prior to exposure (to restraint or isolation in the novel arena) using a light-dark box paradigm, (2) 1 day after the 3-day restraint/isolation protocol ended using a light-dark box paradigm, and finally (3) 2 days after the 3-day restraint/isolation protocol ended using a marble bury test. These tests revealed that 2-h of jacket restraint on each of 3 consecutive days induced persistent elevations in mouse anxiety related behaviors that are indicative of stress (Figure 3). We found a main effect of context on the proportion of time animals spent in the dark in the light-dark box [*F*_(3, 29)_ = 4.6, *p* = 0.010]. In the pre-test session, the eight animals in each of the isolation and jacket restraint groups spent similar proportions of time in the dark (Figure 3A, *p* = 1), indicating that these animals had similar baseline anxiety levels. One day after the 3-day restraint or isolation protocol ended, we found that animals undergoing restraint spent a significantly greater proportion of time in the dark (*p* = 0.030). There was no corresponding change in the behavior of the isolation group (*p* = 1). Two days after the final day of restraint, animal anxiety levels were reassessed using the marble burying test. Mice who underwent restraint buried significantly more marbles (*p* < 0.001) than control animals, indicating that the behavioral effects of restraint persisted at least 2 days after restraint (Figure 3B).

### Vocal behavior

#### Calling rate

The rate at which the mice vocalized depended on the context and number of exposures to the context. Only animals for which all emitted vocalizations were tagged are included in the analysis of calling rate (Number of animals: mating *n* = 14, isolation *n* = 7, restraint *n* = 7); this comprised 60,571 of the total data set of 80,579 analyzed vocalizations. The number of vocalizations emitted within day 1 of a context was highly variable (mating: Mean = 3685, *SD* = 2096, Range = 247–8371; isolation: Mean = 375, *SD* = 391, Range = 21–1043; restraint: Mean = 202, *SD* = 145, Range = 104–479), but differed among contexts [*F*_(2, 26)_ = 17.3, *p* < 0.001]. Animals were dramatically more vocal during mating encounters than in either isolation or jacket restraint (*p* < 0.001 for both). Mice vocalized substantially less during their second and third exposures to isolation in the test arena, averaging 75 vocalizations (*SD* = 131) on the second day and only 47 vocalizations on the third (*SD* = 35). In contrast, there was a minimal change in the number of vocalizations emitted by mice during restraint over the 3 days (day 2 = 345, *SD* = 315; day 3 = 217, *SD* = 167).

For all three contexts, calling rate declined within a 1-h recording session. Calling rates were computed in 10-min time bins for each animal within each context on Day 1. Overall, animals within the mating context vocalized at much higher rates than animals in isolation or restraint (*p* < 0.001 for both comparisons), emitting vocalizations at an average rate of 1.02 Hz (*SD* = 0.82). Within the mating context, the average rate of calling reduced as a function of time from 1.9 Hz initially to 0.6 Hz and there was a strong negative correlation between time bin and calling rate (−0.52, *p* < 0.001). For animals in isolation the average rate of calling reduced as a function of time from 0.16 to 0.04 Hz, with a moderate negative correlation (correlation = −0.3, *p* = 0. 039). There was also a moderate negative correlation for animals in restraint (correlation = −0.3, *p* = 0.006); the average calling rate reduced from 0.13 to 0.03 Hz.

#### Frequency characteristics of vocalizations

**Figure 4:**
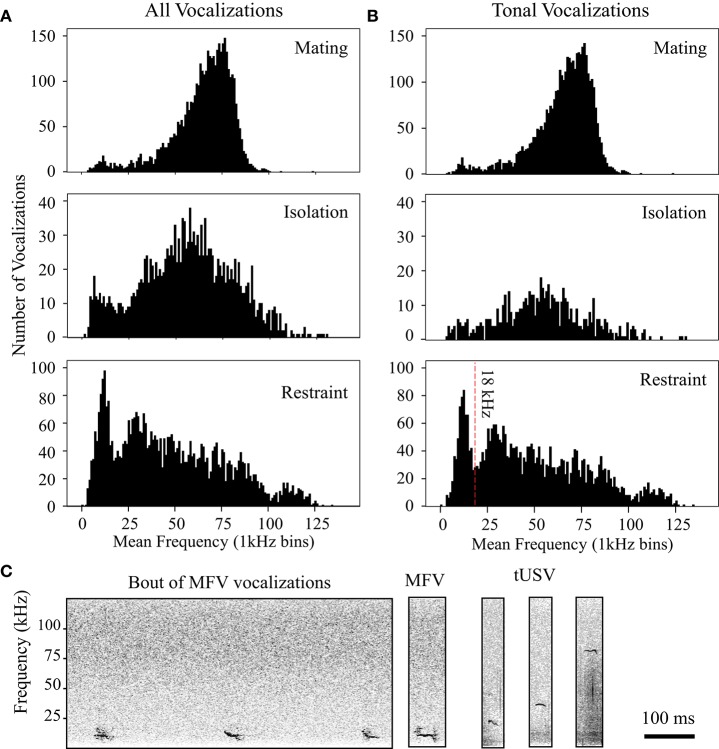
**Distribution of peak frequencies in vocalizations changes with context. (A)** Histograms display peak frequencies of all vocalizations emitted in each context. **(B)** Histograms show distribution of tonal vocalizations emitted in each context. Vertical red line at 18 kHz in restraint histogram indicates criterion used to delineate Mid-Frequency Vocalization (MFV) from tUSVs. All histograms utilize 1 kHz bins. **(C)** Left, spectrograms of mid-frequency tonal vocalizations (MFVs) emitted by restrained mice. Right, MFV, and tUSVs emitted by same animal during restraint.

The frequency distribution of vocalizations changed dramatically with context. Figure 4A shows the mean dominant frequencies of the first 100 vocalizations emitted by all animals on the first day of mating, isolation, or restraint. Compared to vocalizations in the mating interactions, there was a much broader distribution of mean dominant frequencies from the same animals when in isolation or restraint. Note the substantial increase in vocalizations at lower frequencies in these contexts.

As we manually tagged the total 80,579 vocalizations (from *n* = 41 animals), we noted that many tonal vocalizations were emitted within the investigators' audible range (<20 kHz). Figure 4B shows the frequency space occupied by tonal vocalizations in different contexts. In both the mating and isolation contexts, the frequency distribution of tonal vocalizations was not normal (Shapiro-Wilk *p* < 0.001 for both, mating mean = 64.6 kHz, *SD* = 14.5, skewedness, −0.88, kurtosis = 1.89; isolation mean = 55.5 kHz, *SD* = 20.1, skewedness, 0.06, kurtosis = 0.623), and mostly within the ultrasonic range. In both the mating and the isolation contexts, these distributions were not bimodal (coefficient of bimodality, mating = 0.424, isolation = 0.276). However, in the jacket restraint context, there was a bimodal distribution of mean dominant frequency (Shapiro-Wilk *p* < 0.001, mean = 48.8 kHz, *SD* = 31.7, skewedness, 0.701, kurtosis = −5.15; coefficient of bimodality, 0.6). Separating the population at 18 kHz, we identified a new category of tonal mouse vocalization, termed *mid-frequency vocalization* (MFV), that appeared distinct from the tUSV category in spectrum (Figure 4B). The average mean dominant frequency of MFVs emitted by animals in restraint was 12 kHz (*SD* = 3 kHz). It is also noteworthy that the distribution of tUSVs emitted when animals were restrained is substantially broader than during mating or isolation (Figure 4B). Thus, the standard deviation of the mean frequency of tUSVs during restraint was more than double the standard deviation during mating (14 kHz vs. 29 kHz), although the mean frequency of tUSVs during mating was only a few kHz different for these contexts (mating = 65 kHz, restraint = 57 kHz). Many tUSVs emitted during restraint were above 100 kHz, the presumed upper limit of mouse hearing (Müller et al., 2005). Figure 4C displays the broad frequency range of tonal vocalizations recorded from a single animal in restraint. Some example MFVs are provided for download in the Supplementary Material (Supplementary Sound Files 1–3) along with some LFHs for comparison (Supplementary Sound Files 4, 5). Some of these sounds have frequencies near the top end of the human hearing range, with bandwidths that are four times greater than the human hearing range. Within the Supplementary Sound Files, the original sound is presented first, followed by an exemplar that has been reduced in frequency four-fold and elongated in time four-fold.

**Figure 5:**
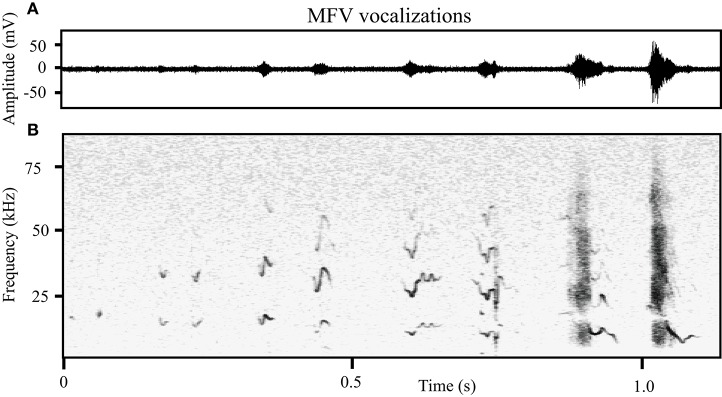
**The structure of MFVs differs with increasing duration. (A)** The relative amplitude of MFVs with different durations recorded from one animal. **(B)** Spectrograms of MFVs with different durations recorded from one animal. Low amplitude MFVs are typically short and have no harmonics. As the amplitude increases, MFVs increase in length and typically have more harmonics.

MFVs often had no harmonics (31%), however the harmonic structure and amplitude of MFV vocalizations differed with duration. Longer vocalizations were emitted at greater amplitudes and with more harmonics (Figure 5). This was represented by a moderate positive correlation between the duration of the vocalization and the amplitude of its fundamental frequency (Pearson's correlation = 0.36, *p* < 0.001). Further, one-way ANOVAs revealed main effects of the number of harmonics within a MFV and the vocalization's duration [*F*_(4, 1964)_ = 18, *p* > 0.001]. The mean duration of MFVs with no harmonics was 26 ms (*SD* = 16 ms) whereas for MFVs with one harmonic it was 34 ms (*SD* = 26 ms, *p* = 0.005). Each additional harmonic corresponded to a significant increase in duration (*p* < 0.001 of each); MFVs with 4 harmonics had mean durations of 37 ms (SD + 26 ms).

A one-way ANOVA also revealed that the amplitude of the fundamental is related to the number of harmonics [*F*_(4, 1964)_ = 269, *p* < 0.001]; MFVs with more harmonics had fundamentals with greater amplitudes. Each additional harmonic corresponded to a significant (*p* < 0.001 for each) increase in the amplitude of the fundamental.

#### Contextual changes in vocal repertoire

**Figure 6:**
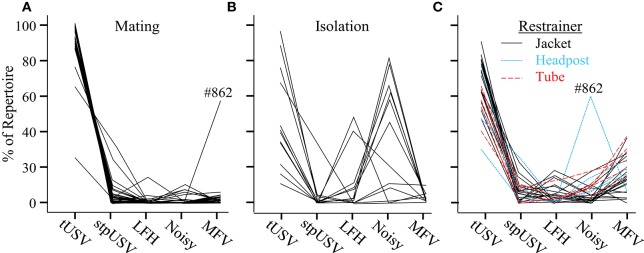
**Proportions of vocal categories differ among contexts**. Each line plots the proportions of vocal categories for one mouse, based on the first 100 emitted vocalizations. Each mouse provided data in **(A)** mating, and in either **(B)** isolation, or **(C)** restraint contexts. One animal, #862, exhibited unusual vocal behavior during both mating and restraint.

The proportions of emitted vocalization types differed strikingly across contexts. Figure 6 displays values for individual animals, calculated using the first 100 vocalizations from each animal (mating, *n* = 28; isolation, *n* = 14; restraint, *n* = 14) on the first day of each context. As previously mentioned, calling rates were substantially lower during isolation and restraint than during mating. Every animal emitted over 100 vocalizations within a 2-h time period during the mating and restraint contexts. In the isolation context, however, 7 of 14 animals emitted fewer than 100 vocalizations during the 2-h period (*n* = 10, 15, 20, 21, 47, 47, 72). Overall, most animals emitted similar proportions of vocal categories within a particular context.

A two-way ANOVA revealed a significant main effect of vocal category on the probability of its emission [*F*_(4, 7995)_ = 6.1, *p* = 0.014], indicating that vocal categories were not emitted with equal probabilities. There was a significant interaction between vocal category and context [*F*_(8, 7995)_ = 78.1, *p* < 0.001]. This indicated that the probability of a vocal category was affected by context. tUSVs were significantly more common during mating (92%) than during isolation (39%) or jacket restraint (66%; *p* < 0.001 for both comparisons). Noisy vocalizations, emitted almost exclusively by isolated animals, were significantly more likely to be emitted during isolation (43%) than during mating (1%) or restraint (8%; *p* < 0.001 for both comparisons). The newly identified MFV vocalization was statistically more likely to be emitted during restraint (15%) than during mating (2%) or isolation (6%; *p* < 0.001 for both comparisons). There was no effect of context on the proportions of LFH or stpUSV vocalizations emitted. The proportional repertoire emitted by animals undergoing restraint was robust over the 3 days of recording and over time within a session, but there were some changes in the repertoire of isolated animals (see Appendix B).

#### Restraint type

To ensure that the emission of MFVs was not an artifact caused by the jacket restrainer, we also recorded vocalizations emitted during tube restraint and headpost restraint (Figure 6C). There was no effect of restraint type on the proportions of vocal categories (*p* = 0.5). Figure 6 shows the proportions of each vocal category emitted by these animals; MFVs were emitted in similar proportions across restrainer types. These data indicate that MFVs are unlikely to result from a particular form of restraint.

#### Vocalizations in bouts

We used data from all animals on all recording days to compare bout structure across contexts. A bout was defined to include at least 3 vocalizations produced successively with silent intervals less than 1569.8 ms (Grimsley et al., 2011). Across contexts, there were differences in the likelihood that vocalizations were emitted within bouts. During mating, the vast majority of vocalizations were emitted within bouts (84%), whereas many fewer vocalizations were emitted in bouts during isolation (27%) or restraint (41%). The number of vocalizations within a bout also differed among contexts [*F*_(2, 4801)_ = 54.7, *p* < 0.001]. During mating, mice emitted 17 vocalizations per bout (*SD* = 27.8), significantly more than within isolation (7.6, *SD* = 32.8) or restraint (7.3, *SD* = 15.7; *p* < 0.001 for both comparisons).

An examination of the third order transitional probabilities within bouts revealed that the vocal repertoire was highly repetitive during mating; there was only a 0.10 probability of transitions between vocal categories. During restraint, however, there was a more than a three-fold increase in the probability of such transitions (0.34). During isolation, the probability of transitions was intermediate (0.16). Within mating, the most common three-vocalization sequence within a bout was tUSV-tUSV-tUSV, representing 85% of triplets, followed by tUSV-stpUSV-stpUSV (3%) and stpUSV-tUSV-tUSV (3%). In isolation bouts, the most likely triplets were: tUSV-tUSV-tUSV (40%), Noisy-Noisy-Noisy (33%) and LFH-LFH-LFH (9%). During restraint bouts, the tUSV-tUSV-tUSV triplet was most common (54%), followed by tUSV-MFV-MFV (13%) and MFV-tUSV-tUSV (13%). MFV-MFV-MFV triplets were also fairly common during restraint (9%; Figure 4C, left).

#### Vocalization duration

**Figure 7:**
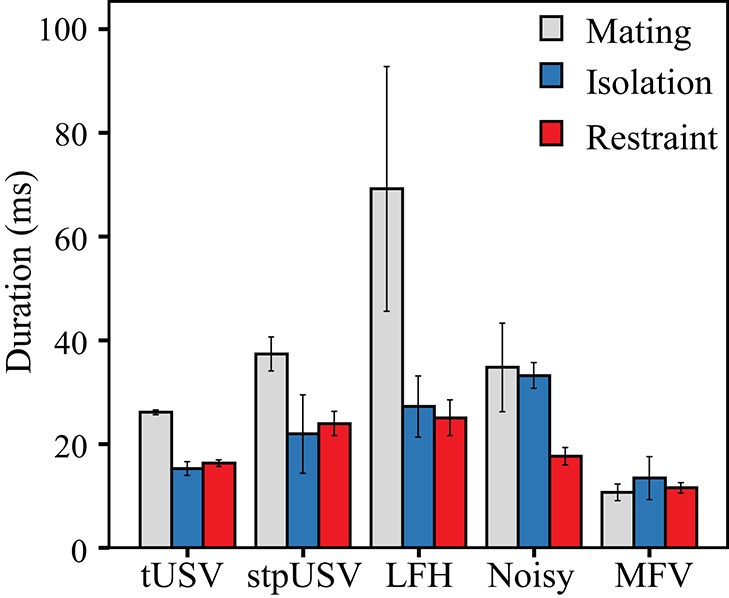
**Mean duration of vocal categories across contexts**. USVs and LFH vocalizations were longer in duration in mating context.

Vocalizations emitted during mating were typically longer than those emitted in isolation or restraint (Figure 7). Across vocal categories, there were main effects of context [*F*_(2)_ = 9.2, *p* = 0.007] and of vocal category [*F*_(4)_ = 4.5, *p* = 0.032] on the duration of vocalizations. There was also a significant interaction between context and vocal category on duration, indicating that the duration not only differs among vocal categories, but that duration is further affected by context [*F*_(8)_ = 30.8, *p* < 0.001]. Thus, tUSVs, stpUSVs, and LFH vocalizations were significantly longer when emitted during mating (*p* < 0.001). The duration of MFVs was significantly shorter than tUSVs (*p* < 0.001). MFVs were of similar durations across contexts.

There was no effect of recording day on the spectrotemporal features of vocalizations (duration, mean dominant frequency, or bandwidth). Only one vocal category, stpUSVs, had durations that were affected by the amount of time an animal had spent within a context. These vocalizations increased in duration within a mating interaction. See Appendix B for details.

## Discussion

The principle goal was to develop a better understanding of how internal state in the form of stress is expressed in the vocalizations of mice. This study compared the vocal behavior of stressed mice with vocal behavior during mating or isolation. This goal aims both to contribute to an understanding of mouse vocal behavioral, as well as to provide better tools by which to analyze mouse models of disease. The study demonstrated that both vocalization repertoire and vocalization structure differ substantially among the tested behavioral contexts. Furthermore, mouse vocalizations during restraint include an audible vocalization that has not been described previously. These results strengthen our understanding of how the internal state of a mouse is revealed by the spectrotemporal features of its social vocalizations.

We also assessed how brief repeated restraint, commonly used in neurophysiological studies to record from unanesthetized animals, affects levels of stress. Our data indicate that even 2-h of restraint for 3 consecutive days induces stress/anxiety in mice that persists for at least 2 days. We also showed that a single 2-h period of restraint is stressful to mice. Our finding that 2-h of restraint is stressful to mice and increases their corticosterone levels is in agreement with prior research in rats showing that 1 h of restraint increases blood corticosterone levels (Mitsushima et al., 2006). These results will aid in our interpretation of subsequent neurophysiological results.

### Contextual effects on mouse vocal behavior

Adult mice vocalize over a broad frequency range from 1 kHz to at least 100 kHz. These vocalizations form several categories; USVs, LFHs, Noisy vocalizations and MFVs. Analyses of adult mouse vocalizations typically focus on vocalizations in the USV category emitted during mating interactions (Scattoni et al., 2009). Emitted by males (Whitney and Nyby, 1979; Maggio et al., 1983), USVs reduce the number of aggressive behaviors from females, induce approach behavior, and facilitate in mating (Sewell, 1969; Pomerantz et al., 1983). Females are attracted to the playback of USVs and to vocalizing males (Pomerantz et al., 1983; Chabout et al., 2015). However, USVs are also emitted in other contexts by both males and females, and the functions and features of these vocalizations in these behavioral contexts are poorly understood. Further, there have been very few studies of the other vocal categories. LFH vocalizations are emitted by male mice who are in pain (Whitney and Nyby, 1983), during handling (Whitney, 1969), and while fighting (Houseknecht, 1968; Gourbal et al., 2004). We and others have shown that structurally similar LFH vocalizations are emitted by females during mating and distress (Sales, 1972a; Grimsley et al., 2013).

Our results reveal several differences in vocal behavior when mice are placed in different behavioral contexts. They preferentially emit different vocal categories, vary the spectrotemporal characteristic of individual vocalizations, and modify the patterns in which these vocalizations are emitted. These differences combine to form vocal behavior that is grossly different among behavioral contexts. In summary:

*Vocal behavior during mating*: Within a mating context, male mice emit long bouts of vocalizations comprising an average of 17 vocalizations. These are emitted at a high rate throughout the mating encounter. The individual vocalizations are emitted at ultrasonic frequencies (between 50 and 100 kHz) and are composed mostly of repeated tUSVs with a few stpUSVs. Under these conditions, males emit very few vocalizations of other types.
*Vocal behavior during isolation*: Animals vocalize at a substantially lower rate in isolation than when they are in a mating interaction. Male mice undergoing isolation typically emit single vocalizations, although they also emit short bouts of vocalizations comprising seven vocalizations on average. tUSVs represent less of the repertoire (39%) and are emitted at lower frequencies and shorter durations than during mating. Noisy vocalizations are very common during isolation (43% of the vocalizations). In fact, they seem to be characteristic of isolation. They are highly unlikely to be emitted by an animal in a mating context (1%) or during restraint (8%).
*Vocal behavior during restraint*: Animals vocalize at a substantially lower rate under restraint than during mating interactions. Vocalizations by restrained mice are emitted more often as single vocalizations rather than bouts of song. Emitted bouts are less repetitive than in mating or isolation, typically comprising seven vocalizations. Similar to vocalizations in mating and isolation, the most common triplet of vocalizations are bouts of three tUSVs. However, these tUSVs emitted by restrained mice have a broader frequency range than during mating (18–125 kHz) and are shorter in duration. MFV vocalizations are characteristic of restraint, representing 15% of the restraint repertoire, and are rarely emitted in other contexts.

### Contextual modulation of mouse vocal categories

The behavioral relevance of USVs is unclear because they are emitted in both positive and negative contexts (Arriaga, 2014). Very few studies have recorded vocalizations emitted in a context other than mating (These include: Maggio and Whitney, 1985; Stowers et al., 2002; Gourbal et al., 2004; Panksepp et al., 2007; Grimsley et al., 2011; Chabout et al., 2015). Only one previous study has investigated contextual effects of restraint on USVs (Chabout et al., 2015).

#### Contextual modulation of MFV

We identified a new category of vocalization in mouse that is commonly emitted during stress. To our knowledge this vocalization has not been reported in other ultrasonic vocalizing rodents. This MFV is emitted within the human hearing range and is typically a quasi-constant frequency vocalization between 9 and 15 kHz with durations between 2 and 43 ms. The MFV was commonly emitted under all tested types of restraint. It is likely that the presence of the MFV has not been previously reported for several reasons. First, as we report, it is very rarely emitted during mating interactions, which is the most common method for collecting adult vocalizations. Second, many studies utilize a high pass filter when analyzing adult vocal data to reduce the interference of high amplitude noise that is generated at low frequencies when animals move through bedding (20 kHz: Nakagawa et al., 2012; 35 kHz: Chabout et al., 2015; 35 kHz: Hammerschmidt et al., 2015). These filters would remove the MFV from analyses. Spectrally, the MFV is similar to the 22 kHz vocalization of rat. The 22 kHz rat vocalization is emitted by animals in situations such as; pain (Tonoue et al., 1986), in response to predators (Blanchard et al., 1991), and aggression (Miczek et al., 1991, 1995). Further, playback of this vocalization elicits defensive behavior from rats (Brudzynski and Chiu, 1995). We hypothesize that playbacks of the MFV vocalization would be behaviorally aversive to mice.

#### Contextual modulation of noisy vocalizations

Noisy vocalizations are multi-harmonic, frequency modulated vocalizations with a noisy warbled harmonic structure not typically included in analyses of mouse vocalizations. This vocalization is rarely emitted during social interactions and is not emitted by pups (Grimsley et al., 2011). However, the present study shows that this vocalization is common in the repertoire of isolated animals. This broadband vocalization includes some exemplars with fundamentals that cover nearly the entire mouse hearing range. This vocalization may be used as a seeking call; its broadband nature would make it ideal for sound localization.

#### Contextual modulation of LFH vocalizations

Here we demonstrate that mice emit a higher proportion of LFH vocalizations during stress. LFH vocalizations emitted during restraint and isolation were shorter in duration than those emitted in mating. Within our previous study, vocalizations were recorded from females during agitation and mating. Here, the longer LFH vocalizations emitted during mating are likely emitted by females and are of similar duration to those recorded earlier (Grimsley et al., 2013). The LFH vocalizations recorded during isolation and restraint were emitted by males and were shorter in duration. These differences could be either a sex difference or contextual in origin. We have shown that males use contextual cues from other modalities to determine whether they should approach or avoid playbacks of the same female LFH vocalization (Grimsley et al., 2013). LFH vocalizations emitted by mice in response to a foot shock are innately aversive to listeners (Chen et al., 2009). Thus, it is likely that the LFH vocalizations emitted by restrained mice serve as distress calls.

#### Contextual modulation of USVs

During social encounters mice typically emit more USVs when they are in physical contact with one another (Ferhat et al., 2015). Several studies have investigated how different contexts affect the emission of USVs in mice. Scattoni et al. (2011) demonstrated that social context (same-sex interactions or mating) does not alter the proportion of different USV subtypes emitted in C57BL/6J and BTBR T+tf/J mice. However, the calling rate of female mice has been shown to change in same sex interactions depending on the estrus cycle (Moles et al., 2007). Here, we demonstrate that the calling rate of mice differs depending on the context (mating, isolation or restraint) and that the proportion of the different USV types emitted differed among contexts. In our previous analysis of CBA/CaJ vocal behavior we, like Scattoni et al. (2011), found no difference in the proportion of syllable types emitted in same sex or mating interactions (Grimsley et al., 2011). It is likely that the difference we see here is due to comparing social with non-social contexts. A recent study compared male mouse USVs (Foxp2-R552H and Foxp2-S321X strains) emitted under three contexts, one of which was non-social and two were social (in response to a cotton swap soaked in water, in response to a cotton swab soaked with female urine and in mating interactions; Gaub et al., 2016). USVs emitted by animals in response to the water soaked swab, which is somewhat similar to our isolation context, were higher in frequency and shorter in duration than those emitted in response to urine. Similarly, we have shown that tUSVs emitted by isolated mice are shorter than those emitted by the same mice in mating interactions. However, we show that isolated males emit USVs that are typically lower in frequency than animals in mating interactions; this difference may be due to the different inclusion criterion for vocalizations as Gaub et al. (2016) excluded data below 20 kHz.

Comparing features of mouse USVs across studies is difficult because researchers use grossly different categories to separate USVs. The one category that is fairly stable across studies includes USVs with frequency steps. Comparisons with other vocal categories is more difficult. For example, different studies have used a wide range of maximum durations to characterize a USV as being “short,” from 5 ms (Scattoni et al., 2008, 2009; Grimsley et al., 2011; Nakagawa et al., 2012) or 10 ms (Branchi et al., 1998), up to 50 ms (Enard et al., 2009; Chabout et al., 2012). Varying this inclusion criterion results in short vocalizations making up a small proportion of the USV repertoire (Scattoni et al., 2008; Grimsley et al., 2011) or the majority (Enard et al., 2009; Chabout et al., 2015). To put this into perspective, applying these different criteria to the present data results in percentages of short vocalizations ranging from 1.6 to 75.1% of the 80,579 vocalizations. To our knowledge, the only prior study to examine vocalizations emitted by isolated and restrained mice compared those data with vocalizations emitted during male-male social interactions (Chabout et al., 2012). That study separated the USV category into 10 subtypes, but used a wide category for inclusion within their short USV category (all vocalizations with no frequency steps with durations less than 50 ms). Because the inclusion criteria for the short USV category was so broad, this USV group was the most common across all contexts. Because of its wide inclusion criteria, it corresponds most closely to our tUSV category. The other three vocal categories we analyzed—MFV, Noisy and LFH—were not included within their study as their focus was on USVs. In agreement with Chabout et al. (2012) we found context dependent differences within the repertoire of USVs and showed that the spectrotemporal characteristics of USVs without frequency steps differed among social interactions, isolation, and restraint. Mun et al. (2015) recently demonstrated that C57/BL6 mice exploring novel environments emit vocalizations at lower frequencies when the environment is more negative. Direct comparisons with the MFV are not possible as vocalizations were not separated into subcategories and no spectrograms or exemplars were provided. We provide further support for the finding that animals in stressful situations emit tUSVs that are lower in frequency and shorter in duration than when animals are in a social situation. Beyond this, we found distinctive changes in the emission pattern of USV that was dependent on the context.

Chabout et al. (2012) demonstrated that mice who are isolated or restrained are unlikely to emit stpUSVs. We also found low emission rates for stpUSVs during restraint and isolation. Within a mating context stpUSVs have been shown to be more likely to be emitted around periods of mounting, whereas tUSVs are more common as a male approaches a female prior to mounting (Wang et al., 2008). Here we observed no change in the proportion of stpUSVs emitted over time, nor did we see a change in the number of frequency steps. However, the duration of the stpUSV did increase over a 1 h mating interaction. We did not analyze the behavior of the male mouse during mating so it is likely that the data here are pooling periods of chasing and attempted mounting across time and across animals. This would likely wash out the effects others have seen on the increased probability of emitting stepped USVs during mounting phases.

### Context-dependent vocalizations in rodents: Comparison to rat vocalizations

Rats emit vocalizations in two functional categories that have distinct spectrotemporal properties. Both are considered to be USVs. The “50 kHz” category is composed of several vocal categories emitted in complex sequential patterns similar to mouse USVs, and are emitted at frequencies between 32 and 96 kHz (Portfors, 2007). The temporal structure is similar to mouse USVs. The 50 kHz USV is emitted during positive behavioral contexts that include social interactions (Blanchard et al., 1993), play (Knutson et al., 1998), and mating (McIntosh and Barfield, 1980). It does not appear to be emitted in the wide range of contexts that mice emit USVs. The lower frequency “22 kHz” category is composed of a tonal vocalization emitted between 18 and 32 kHz (Portfors, 2007). This vocalization is emitted by animals in aversive situations that include foot shock, fear, and in response to predator cues (Tonoue et al., 1986; Blanchard et al., 1991; Miczek et al., 1991, 1995). Of particular interest, rats emit the 22 kHz signal during restraint, and the rate of calling in male rats reduces over time during restraint (Mitsushima et al., 2006). The two rat USV types elicit distinct responses among neurons of the basolateral amygdala (Parsana et al., 2012), a brain region associated with emotional responses to sensory stimuli, including social signals (Gadziola et al., 2012; Grimsley et al., 2013).

There has been no observed structural counterpart in mice to the rat 22 kHz USV, however the MFV vocalizations that we identified has some similarities. It is lower in frequency than other types of vocalizations and it is emitted in stressful contexts that include restraint. The temporal patterning is quite different, in mice MFVs are emitted singly or at low rates, whereas the rat 22 kHz vocalizations are emitted repeatedly at high duty cycle. Mice also emit other vocalizations under stressful conditions, including USVs and the LFH vocalizations.

#### The effects of restraint on mouse corticosterone levels and behavior

Although stress has been shown to affect the perception of sound in humans (Muchnik et al., 1980; Horner, 2003; Cromwell and Atchley, 2015), restraint is typically used in neurophysiological studies when recording responses to vocalizations and other sounds from unanesthetized rodents (Philibert et al., 2005; Portfors et al., 2009; Lin and Liu, 2010; Muniak et al., 2012; Duque and Malmierca, 2015). Many researchers advocate repeated exposure to restraint prior to electrophysiological recordings in order to habituate the animals to restraint. However, both acute and repeated restraint induce stress in mice and are commonly used to assess the effects of stress on mice (for review see Sutanto and Dekloet, 1994). Furthermore, restraint affects inhibition within the auditory system and alters responses to sound in the rodent auditory cortex (Pérez et al., 2013; Ma et al., 2015). Our findings provide further evidence that both brief and repeated restraint, similar to that used in electrophysiological studies, are stressful to mice. We propose that restraint stress will also affect the neural representations of vocalizations and that the recording context should be taken into account when interpreting findings.

## Conclusion

This work demonstrates that mouse vocalizations vary with the behavioral context of the calling mouse. USVs, the dominant vocalization type in the mouse repertoire, undergo substantial changes in frequency, duration, and calling rate when emitted during isolation or restraint, compared to USVs emitted in mating. This work also demonstrates that non-USV vocalizations are produced in significant numbers in more negative contexts. The newly described MFV is emitted primarily during restraint, while the noisy call is emitted preferentially in isolation. This work suggests a greater role for the non-USV calls in mouse communication than has been recognized previously. Overall, the study reveals vocal behavior that is grossly different among behavioral contexts and may reflect the level of anxiety in these contexts.

## Author contributions

JG–Designed experiments, collected data, designed analysis, analyzed data, wrote the manuscript. SS–Collected data, analyzed data, helped edit the manuscript. NV–Collected data, analyzed data, helped edit the manuscript. CG–Collected data, analyzed data, helped edit the manuscript. JB–Collected data, analyzed data, helped edit the manuscript. ML–Collected data, analyzed data, helped edit the manuscript. AJ–Analyzed data, helped edit the manuscript. JW–Designed experiments, designed analysis, wrote the manuscript.

## Funding

This study was supported by National Institute on Deafness and Other Communication Disorders Grant R01 DC-00937-25 to JJW.

### Conflict of interest statement

The authors declare that the research was conducted in the absence of any commercial or financial relationships that could be construed as a potential conflict of interest. The reviewer AR and the handling Editor declared their shared affiliation, and the handling Editor states that the process nevertheless met the standards of a fair and objective review.
