# Supplementary material for: Contextual Modulation of Vocal Behavior in Mouse: Newly Identified 12 kHz “Mid-Frequency” Vocalization Emitted during Restraint
Source: Front Behav Neurosci. 2016 Mar 9;10:38. doi: 10.3389/fnbeh.2016.00038 (PMC4783392; doi:10.3389/fnbeh.2016.00038)
Supplement: Supplementary file 1 [file Presentation1.PDF]

Supplementary Information

Appendix A: Repertoire is generally consistent within and across sessions.

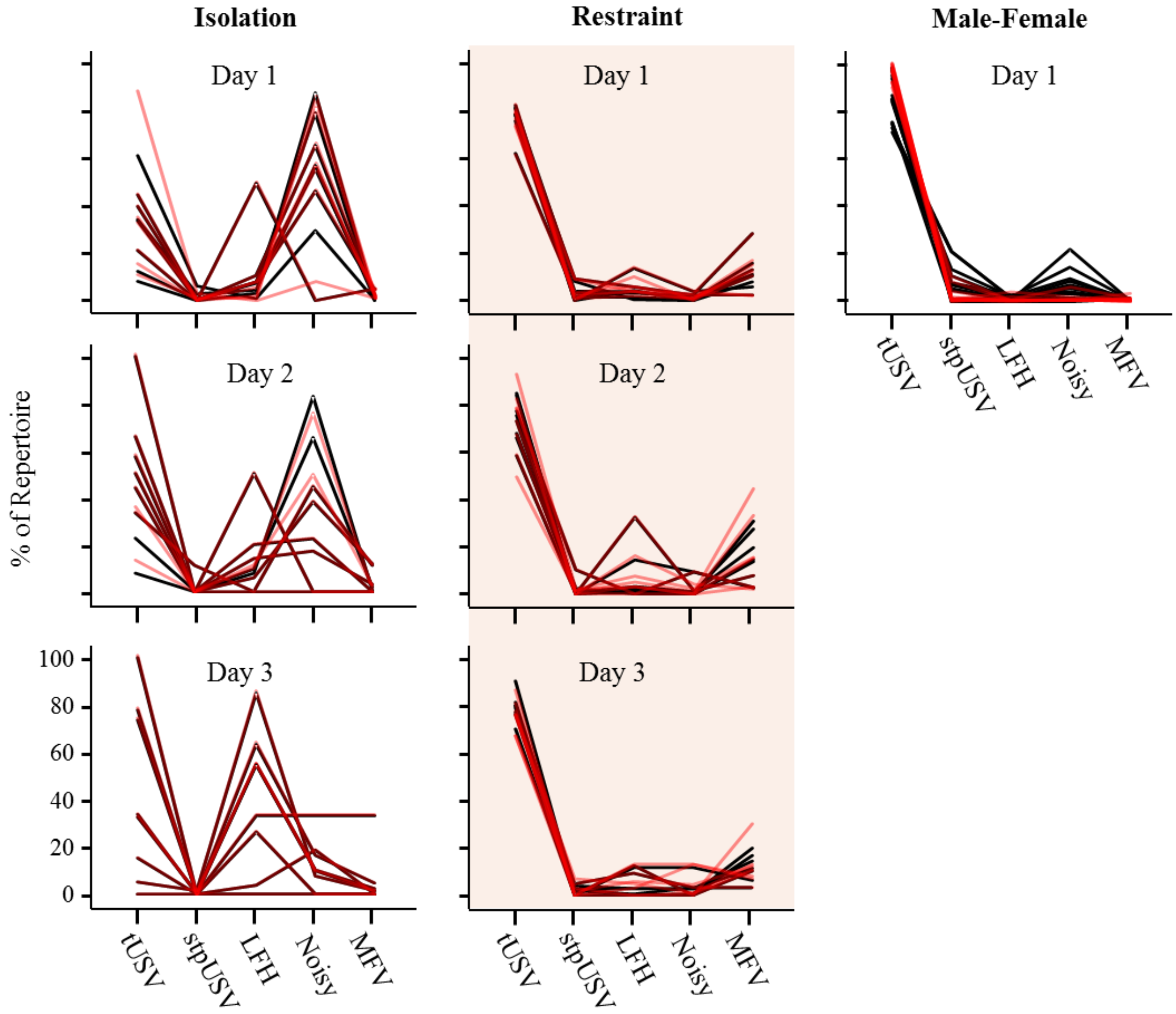

Supplementary Figure 1. Comparison of vocal repertoire within and across sessions. Within each graph, each animal's repertoire is compared based on all vocalizations (black lines) versus the first 100 vocalizations within a session (red line). Note close correspondence (overlap) between the two measures in most cases. This figure also compares vocal repertoire across sessions for isolation and restraint. Note change in repertoire in isolation between Day 2 and Day 3.

The first 100 vocalizations emitted were generally representative of the repertoire for all three contexts: mating, isolation, and jacket restraint (Supplementary Figure 1). A multivariate ANOVA investigated whether there was an effect of analyzing only the first 100 emitted vocalizations on the repertoire. There was no main effect of using all or just the first 100 vocalizations ($p=1$), nor was there an interaction with context ($p=1$) or vocal category ($p = 0.5$). There was also no interaction among all three. This indicates that the first 100 vocalizations within a session provides a representative sample of the vocal categories emitted within each context. There was an interaction between the context and the vocal category ($F (8) = 81$, $p <$

0.001), indicating that vocal categories are emitted with different probabilities within different contexts.

The repertoire emitted by animals undergoing restraint was robust over the three days of recording (n=8), but we observed repertoire changes in mice (n = 8) undergoing isolation between Day 2 and Day 3 (Supplementary Figure 1). A multivariate ANOVA was used to investigate whether several factors affected the observed vocal repertoire, based on the first 100 vocalizations. These factors included vocal category (tUSV, stpUSV, LFH, Noisy, MFV), recording day (1, 2, 3), and context (mating, isolation, jacket restraint). In agreement with our larger sample of animals analyzed during the first day of restraint, we found a main effect of vocal category, indicating that some vocal categories are more prevalent than others ($F(4) = 7.7$ $p < 0.001$). There was a significant interaction between vocal category and context, indicating that the context affected the vocal repertoire ($F(8) = 5.4$ $p = 0.015$). Further, there was a significant interaction among vocal category, context, and day, indicating that some vocal categories are produced in different proportions on some days in at least one context ($F(8) = 7.4$, $p < 0.001$). Follow-up analyses with Bonferroni corrections revealed that the repertoire did not differ for animals undergoing restraint ($p > 0.524$ for all vocal categories). However, animals undergoing isolation emitted a different repertoire on the Day 3. Most animals switched from emitting Noisy vocalizations to emitting LFH vocalizations. There were significantly fewer Noisy vocalizations emitted on Day 3 for isolated animals ($p < 0.05$) and a corresponding significant increase in the proportion of LFH vocalizations emitted on Day 3 ($p < 0.05$).

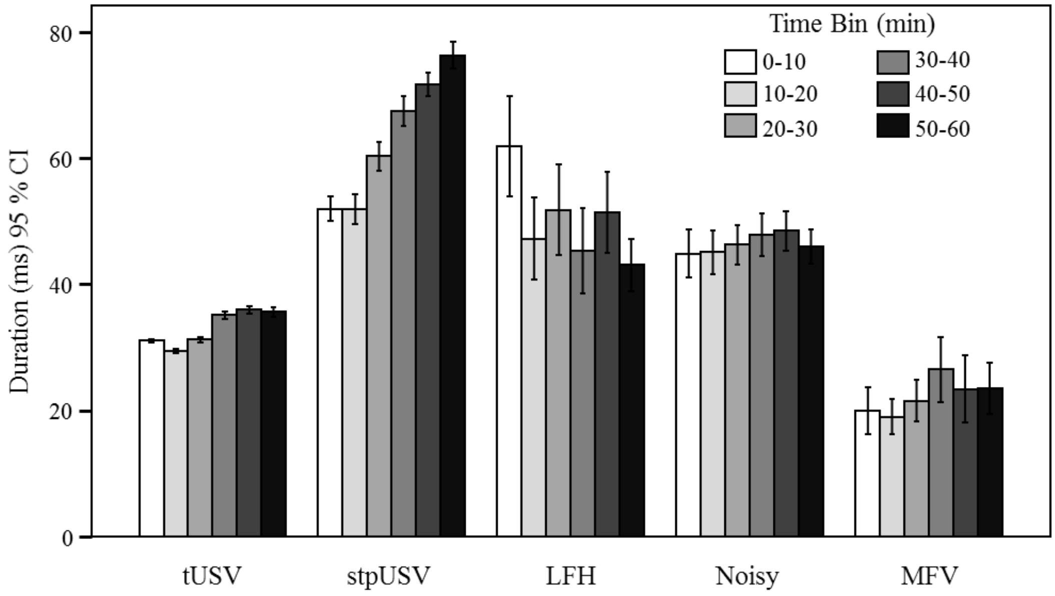

Supplementary Figure 2. Mean duration of vocalizations during a one-hour mating interaction. Mean durations are displayed in 10 min bins. The stpUSV and tUSV categories change significantly during this time, increasing steadily in duration over the one-hour mating period.

Appendix B: Some vocalizations change within sessions. We examined whether the duration of vocalizations changed with recording sessions by comparing differences between the first 100 vocalizations and the entire sample. The stpUSV and the tUSV displayed within-session changes (Supplementary Figure 2). There was a significant interaction between sample type (first 100 or

all vocalizations) and vocal category on the mean duration of vocalizations, indicating that at least one vocal category had a duration that differed. Follow-up analysis with Bonferroni corrections revealed that stpUSVs were significantly longer ($p < 0.001$) when all vocalizations are included rather than just the first 100. Further analysis revealed a correlation between time within the mating interaction and duration of stpUSVs (Pearson's correlation (3427) = 0.34, $p < 0.001$). As shown in Supplementary Figure 2, stpUSVs were initially emitted with durations of approximately 52 ms (SD = 26 ms). However, duration increased to 76 ms (SD = 25 ms) by the end of the session, representing a 46% increase in duration. The tUSV vocalizations emitted within the first 30 minutes (0-10, 10-20 and 20-30 minute time bins) were significantly shorter than those emitted during the second thirty minutes (30-40, 40-50 and 50-60, $p < 0.001$ for all comparisons).

Mice emit stpUSVs with different numbers of steps (Holy and Guo, 2005;Portfors, 2007;Grimsley et al., 2011). We investigated whether within-session changes in stpUSV duration were related to an increase in step number. Across the six time-bins between 0 and 60 mins), we found that the proportion of step number remained the same (1-Step, range 43-45%, 2-Step, range 43-45%; 3-Step range, 11-14%). There was a main effect of step number on the duration of stpUSVs ($F (2, 2443) = 20.4$, $p < 0.001$), such that vocalizations with more steps were longer in duration. However, there was no interaction between the number of frequency steps and the time bin, indicating that all subtypes increase in duration over time (Supplementary Figure 3).

The rate of calling within a session reduced as a function of time (data shown in main text), however, all call types were emitted throughout the first hour of recording within a context. Supplementary figure 4 shows the emission rates for each call type during the first hour in ten minute time bins. These data are from animals where all vocalizations were tagged (Number of animals: mating n = 14, isolation n = 7, restraint n = 7).

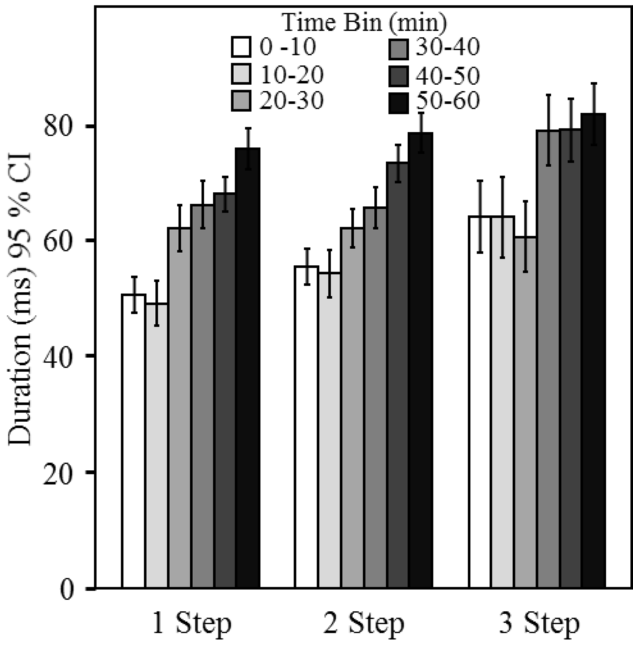

Supplementary Figure 3. Durations of all stepped USVs increases over time. Mean duration of stpUSVs displayed in10 min bins during a one-hour mating exposure. All groups of stpUSVs showed increased duration over time, independent of the number of frequency steps within the vocalization.

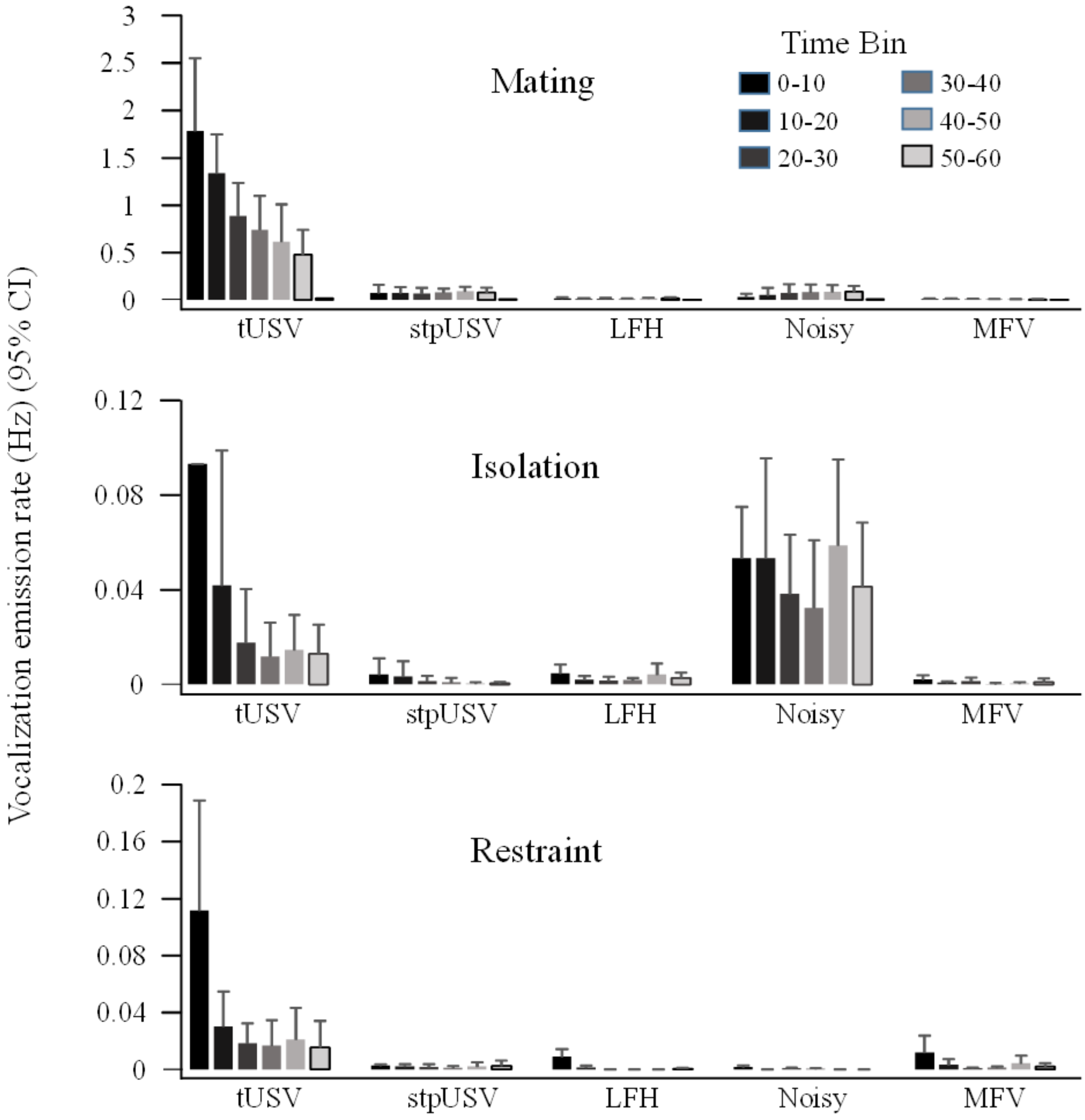

Supplementary Figure 4. Mean emission rate of each call type during the first hour in 10 minute time bins. The emission rate in the mating context was greater than in both the isolation and restraint contexts. The rate of calling reduced over the first hour. All vocalization types were emitted within each time window.

References

Grimsley, J.M., Monaghan, J.J., and Wenstrup, J.J. (2011). Development of social vocalizations in mice. *PLoS ONE* 6**,** e17460. doi: 10.1371/journal.pone.0017460.

Holy, T.E., and Guo, Z. (2005). Ultrasonic songs of male mice. *PLoS Biol* 3**,** e386.

Portfors, C.V. (2007). Types and functions of ultrasonic vocalizations in laboratory rats and mice. *J Am Assoc Lab Anim Sci* 46**,** 28-34.
